# Maternal and neonatal data collection systems in low- and middle-income countries: scoping review protocol

**DOI:** 10.12688/gatesopenres.13106.1

**Published:** 2020-02-05

**Authors:** Mabel Berrueta, Ariel Bardach, Agustin Ciaponni, Xu Xiong, Andy Stergachis, Sabra Zaraa, Pierre Buekens, Scoping Review Collaboration Group

**Affiliations:** 1 Department of Mother and Child Health Research, Institute for Clinical Effectiveness and Health Policy (IECS), Buenos Aires, C1414CPV, Argentina; 2 Argentine Cochrane Center, Institute for Clinical Effectiveness and Health Policy (IECS), Buenos Aires, C1414CPV, Argentina; 3 Tulane University School of Public Health and Tropical Medicine, New Orleans, Louisiana, LA 70112, USA; 4 University of Washington, Seattle, WA 98195-7631, USA

**Keywords:** LMICs, MNCH, active surveillance, electronic registries, maternal vaccines, pregnancy, pharmacovigilance, health information system.

## Abstract

**Background:** Pregnant women and neonates represent one of the most vulnerable groups, especially in low- and middle-income countries (LMICs). A recent analysis reported that most vaccine pharmacovigilance systems in LMICs consist of spontaneous (passive) adverse event reporting. Thus, LMICs need effective active surveillance approaches, such as pregnancy registries. We intend to identify currently active maternal and neonatal data collection systems in LMICs, with the potential to inform active safety electronic surveillance for novel vaccines using standardized definitions.

**Methods:** A scoping review will be conducted based on established methodology. Multiple databases of indexed and grey literature will be searched with a specific focus on existing electronic and paper-electronic systems in LMICs that collect continuous, prospective, and individual-level data from antenatal care, delivery, neonatal care (up to 28 days), and postpartum (up to 42 days) at the facility and community level, at the national and district level, and at large hospitals. Also, experts will be contacted to identify unpublished information on relevant data collection systems. General and specific descriptions of Health Information Systems (HIS) extracted from the different sources will be combined and duplicated HIS will be removed, producing a list of unique statements. We will present a final list of Maternal, Newborn, and Child Health systems considered flexible enough to be updated with necessary improvements to detect, assess and respond to safety concerns during the introduction of vaccines and other maternal health interventions. Selected experts will participate in an in-person consultation meeting to select up to three systems to be further explored in situ. Results and knowledge gaps will be synthesized after expert consultation.

## Introduction

Pregnant women and neonates are two of the most vulnerable groups globally. Despite significant achievements in reducing preventable maternal, newborn, and child morbidity and mortality, in line with the Sustainable Development Goals, further progress is still needed, especially in low- and middle-income countries (LMICs) ^1^.

A systematic analysis for the Global Burden of Disease Study reports that respiratory infections and sepsis are among the leading causes of neonatal deaths in 195 countries across the world ^2^. The development of new vaccines to enhance maternal immunization, e.g. for group B streptococcus and respiratory syncytial virus, have the potential to reduce serious morbidity and mortality in newborns. However, new vaccine development requires robust systems to collect and assess maternal, newborn, and child health (MNCH) outcomes, including safety information, following the use of novel vaccines, drugs, and biological products during pregnancy. Indeed, the US Food and Drug Administration (FDA) and the European Medicines Agency (EMA) require extensive post-licensure safety monitoring commitments, particularly active surveillance, from market authorization holders for health products that could be used during pregnancy ^3^.

A recent analysis, funded by the Bill and Melinda Gates Foundation ^4^, reported that the majority of vaccine pharmacovigilance systems in LMICs consist of spontaneous (passive) adverse event reporting, where any health professional or patient, among other individuals, are able to report a suspected adverse event. In order to interpret the data, however, knowledge of the background rates of health outcomes of interest is needed, as well as data regarding the number of individuals exposed to the vaccine. Mapping existing systems will facilitate the potential for countries to work and increase their ability to monitor the most important health outcomes following immunization ^5^. Background population data on adverse pregnancy outcomes are also helpful for designing active vaccine safety surveillance studies at sentinel sites.

Surveillance systems have been established in certain LMICs for monitoring birth, deaths, and key health indicators. Additionally, the field of vaccine pharmacovigilance has expanded, but it has limited application to maternal immunization vigilance in LMICs. Major challenges are the recording of data across different health system levels using different tools and platforms, different definitions and classifications for maternal and neonatal outcomes, the need for standardized definitions and measurement (e.g. gestational age), and the need for information about exposures, such as vaccines, disaggregation of data, and the applicability of standards to LMICs ^6^.

The Global Alignment of Immunization Safety Assessment in pregnancy (GAIA) project proposed case definitions for key events in MNCH and piloted them in 24 sentinel sites across four World Health Organization (WHO) regions ^7^. A feasibility assessment evaluated the ability of GAIA case definitions to identify and classify selected outcomes and/or maternal vaccination in LMICs. Additional field testing in LMICs of the GAIA definitions is ongoing, as there are continuing questions about their practicality, utility, and impacts on improving data quality ^8, 9^.

In high-income countries, MNCH outcomes are tracked through vital registration systems and data from medical records and other national health reporting systems. In LMICs, however, these data are generally not available, nor do they interface with pharmacovigilance platforms or initiatives. Births, deaths, and clinical events often occur outside of medical facilities; vital registration systems are not always comprehensive; and medical records are often incomplete, poorly maintained and paper-based, making it cumbersome to link these across systems. However, a number of surveys, surveillance systems, and health information systems are in place that could provide information on maternal and infant health in low-resource settings. Thus, we intend to identify existing electronic and mixed paper-electronic systems that collect continuous maternal and neonatal data in LMICs, using a scoping review, with the potential to inform active safety electronic surveillance for novel vaccines using standardized definitions. Given the importance of improving maternal interventions vigilance, the scoping review will also identify active safety surveillance systems in LMICs as applied to MNCH.

## Methods

A scoping review is considered to be the most suitable approach to achieve the broad aim of this study. In contrast to the traditional systematic literature reviews that aim to answer specific questions, scoping reviews produce a broad overview of the field ^10, 11^. Scoping reviews may be undertaken to examine the extent, range, and nature of activities in a particular area, to summarize and disseminate findings, and to identify gaps in the existing body of knowledge. In addition to searching the published literature in the main biomedical databases, grey literature is also explored, since it is very likely that most information (i.e. descriptions of systems that collect continuous perinatal data, pregnancy registries, etc.) may be found in government agency and non-governmental organization websites, international organizations, such as the WHO, countries’ official Ministry of Health reports and technical guidance or regulatory documents, and meeting proceedings, among others.

Drafting and reporting of the scoping review will be guided by the Preferred Reporting Items for Systematic reviews and Meta-Analyses extension for Scoping Reviews (PRISMA-ScR) Checklist ^12^. This scoping review will use the methodological framework proposed by Arksey and O’Malley ^10^, as well as the amendments made to this framework by Levac *et al.* ^13^ and by the Joanna Briggs Institute ^14^. The framework consists of six consecutive stages: 1) identifying the research question; 2) identifying relevant studies; 3) study selection; 4) data extraction; 5) collating, summarizing, and reporting results; and 6) consultation. Each stage is briefly discussed below.

### Stage 1: Identifying the research question

Arksey and O’Malley ^10^ suggest an iterative process for developing one or more research questions. In the first stage, two research questions for this scoping review have been identified based on gaps in the literature:

- What existing prenatal and postnatal data collection systems are in place at the facility level and community level that could provide continuous, longitudinal, and individual information on maternal and neonatal health outcomes in LMICs?
- Do existing prenatal and postnatal data collection systems have the capacity to inform active safety surveillance for maternal vaccines and other maternal health interventions?

### Stage 2: Identifying Relevant Studies

A comprehensive search strategy will be developed in order to identify relevant literature from 2014 or the year of database inception to August 2019, underpinned by key inclusion criteria. These are based on the ‘Population–Concept–Context’ framework recommended by the Joanna Briggs Institute for scoping reviews ^14^, which has roots in the PICO (population, intervention, comparator, and outcome) framework commonly used to focus clinical questions and develop systematic literature search strategies.

***Population–Concept–Context.*** P—Population = Pregnant women and neonates.

C—Concept = Articles with a specific focus on existing electronic and paper-electronic systems, in LMICs that collect continuous, prospective, and individual-level data from antenatal care, delivery, postpartum (up to 42 days), and neonatal care (up to 28 days) at the facility or/and community level, at the national and/or district level, and/or at large hospitals.

From the identified systems, we will record information on the following data points:

a) The country/-ies of operation.
b) The extent of the registry data collection (in facilities only, in community services only, both, or not defined).
c) The primary purpose of the system, such as clinical care information systems, mortality registries, insurance registries, birth registration, other.
d) Records linkage, such as the use of unique, individual identifiers that allow for tracking mother-newborn-child across records and time.
e) The scale of the implementation of the registry (national, district, local, or not defined).
f) The specified population captured by the registry data collection (total population, only subgroups/select population, or not defined).
g) The data collection method used (paper, electronic, both, or not defined).
h) Whether the primary data were collected and entered directly into the registry, or if the registry was based on a secondary/duplicate data collection from existing sources (direct, duplicated, or not defined).
i) Health care periods that the system registers (antenatal care, delivery, neonatal, postnatal care, until facility discharge)
j) Whether it collects pregnancy and neonatal outcomes defined by the GAIA project ( *Extended data*)
k) Whether it collects maternal sociodemographic variables (age, education, etc.), obstetric characteristics (gestation, parity, etc.), pre-existent conditions (HIV, syphilis, other), and process of ANC (number of visits, gestational age at first visit, preventive interventions, vaccine administration, etc.)
l) Type of terminology used (MedDRA, SNOMED, ICD10, other, not defined)
m) Who, where, and how individual data are captured
n) Baseline data (pregnancy and outcomes) timeframe
o) Capacity to compile and transfer electronic data
p) Whether it uses a mechanism/process(es) to link maternal and neonatal health records, laboratory registry, medications, vaccines, other exposures of relevance, and to pool and link to other different data sources (vital statistics, etc.); if yes, description of the mechanism/process
q) Whether and how data are monitored for quality; if yes, description of the mechanism/process(es)
r) Data repositories and back-up policy.
s) Whether data collection tools and system documentation are available
t) How maintenance and updates are performed
u) Data access, data extraction, and the ability to import and export data
v) Infrastructure status and whether the privacy protection is internal and external
w) Sponsor/funding
x) If the system had been used previously for active surveillance or pharmacovigilance or post-marketing surveillance; if yes, what type of post-marketing surveillance used (active vs passive, general vaccines vs maternal vaccines, surveillance of drugs, vaccines, other interventions)

C—Context = The review will include all study designs (e.g., randomized controlled trials, non-randomized comparative, pre-post, survey/cross-sectional, case-control, cohort, qualitative, case studies) and will not have language restrictions.

***Inclusion criteria.***

- Electronic or paper – electronic registries.
- LMICs ( *Extended data*).
- Maternal and Neonatal Information Systems that continuously collect perinatal outcomes during antenatal care, delivery care, childbirth, and neonatal periods.
- Existing system and used in the past 5 years.
- Demonstrated ability to collect prospective, longitudinal, and individual-level data.
- Active surveillance of maternal interventions, including pregnancy exposure registries and other types of active surveillance, including sentinel site active surveillance of maternal interventions.

***Exclusion criteria.***

- Paper-based only.

***Search strategy.*** The search strategies, including search terms, will be initially drafted by the investigator team, then by an experienced librarian; it will be further refined through the team’s review and discussion.

We will run an initial search in MEDLINE. Subsequently, the following databases will be searched: EMBASE, Global Health-OVID, Cochrane, LILACS (BVS-iAH-EN), Index Africanus, Bibliography of Asian Studies (BAS), Index Medicus for the Eastern Mediterranean Region, Index Medicus for the South-East Asian Region, IndMed, KoreaMed, Cumulative Index to Nursing and Allied Health Literature (CINAHL), Educational Resources Information Center, PsycINFO, Scopus and Web of Science ( *Extended data*).

In the third and last step, reference lists of included studies, as well as websites of journals that display a strong interest in perinatal health information systems, as evidenced by numerous publications on the topic, will be hand-searched using keywords related to continuous perinatal health information systems, as outlined in the MEDLINE strategy to identify any additional literature.

The search strategy for grey literature will include searching in websites of existing Health Information Systems (HIS) (i.e., Sistema Informatico Perinatal (SIP), Global Network, DHIS2, INDEPTH, MRC and Wellcome Trust networks, Pasteur networks, etc.). We will also conduct searches of organizations that offer resources to evaluate HIS (Measure Evaluation, WHO/PAHO web, CARPHA, etc.), Ministries of Health of LMICs websites, and the Global Alignment of Immunization Safety Assessment in pregnancy website. We will contact authors to identify additional sources and contact experts to identify unpublished information on relevant data collection systems. Also, a custom Google search will be performed using key words, translating the search strategy developed for literature databases. Finally, we will explore grey literature databases like OpenGray and SIGLE, MNCH meetings and websites of large MNCH projects, e.g., http://www.mcsprogram.org.

The literature search strategy will be reported in a manner that allows easy replication by others and will be presented in its entirety in the text, a table, and/or an appendix. The final reference lists of the articles included will be hand-searched for additional information. If data or data subsets of the same population were published in more than one article, we will select only the publication with the largest sample size, as appropriate.

### Stage 3: Study selection

Following the execution of the search strategy, the identified records (titles and abstracts) will be collected in a reference manager for de-duplication and then uploaded into Cochrane’s COVIDENCE online software to manage the initial phases of scoping review and overviews. This software also enables independent screening and logs disagreements and consensuses among reviewers.

According to the eligibility criteria mentioned above, the studies’ selection processes will be implemented over two stages. The first stage will involve the screening of each title and abstract in COVIDENCE by at least two independent reviewers to determine its eligibility for full-text screening. Each article will be categorized into one of three categories (Yes, Maybe, No) to assess the relevance and probability of full text retrieval.

In the second stage, all articles except those categorized as “No” (excluded) will be retrieved in full text for further analysis. Disagreements between reviewers will be resolved by consensus of the whole team.

An adapted version of the PRISMA flow diagram will be used to report final numbers in the resulting study publication once the review is completed. Reasons for exclusion will be recorded at the full-text review stage.

### Stage 4: Data extraction

A form for data extraction will be developed at the protocol stage to extract and sort key pieces of information from the selected full text articles. It will be pilot-tested and refined during the full-text screening stage in order to capture more detailed information. Additional categories that may emerge during data extraction will be added accordingly. Data extraction will be done using Google documents’ online spreadsheets. A framework to assess and describe the existing MNCH health systems will be created based on some frameworks available in the literature.

### Stage 5: Collating, summarizing, and reporting the results

In order to create a useful summary of the data, we will combine all Perinatal Health Information Systems and their characteristics from all sources into table(s) as appropriate.

A checklist for reporting scoping reviews, the ‘Preferred Reporting Items for Systematic Reviews and Meta-Analysis: extension for Scoping Reviews (PRISMA-ScR),” will be used ( *Reporting guidelines)*.

Data will be organized in a database to create an Evidence and Gap map that will provide a visual overview of the geographical distribution of systems reviewed. The map will identify evidence gaps by comparing the key research questions identified with the available literature. It will be presented as an interactive geographical map and table(s). Analysis will be stratified by Global Alliance for Vaccines and Immunization (GAVI) country so that the results will be stratified by GAVI versus non-GAVI LMICs.

Active surveillance vaccine studies and systems in pregnancy that use MNCH systems that are compliant with the eligibility criteria of this scoping review will be listed as relevant literature to discuss further.

### Stage 6: Consultation

We will organize an Advisory Group meeting in Year 1 in New Orleans, Louisiana, USA, to discuss the results of the scoping review, seek additional expert input, and select up to three systems to be further explored *in situ*.

We will present a final list of existing MNCH systems that were considered to help companies/researchers/regulators understand where to access and abstract population-based background pregnancy outcomes data. This will aid in informing the denominator of active vaccine safety surveillance studies that are flexible enough to be updated, if necessary, to detect, assess, and respond to safety concerns derived from the introduction of novel maternal vaccines.

Based on scoping review results, Advisory Board members will review the information technology platforms from selected existing MNCH systems, especially core pregnancy outcome variables used across the systems, data quality control provisions, their ability to export data, how they are characterized, whether they easily could integrate with minimum requirements for active vaccine safety surveillance to help define the denominator of clinical and case-control or cohort surveillance studies indicator rates, and their compliance with Good Clinical Practice, as appropriate.

## Dissemination

Results of this scoping review will be published in a peer-reviewed journal and presented at conferences, as appropriate. All publications will be submitted to open access journals, and the databases, tools, and other materials generated by this project will be made publicly available.

## Study status

The scoping review is in progress. Stage 1 to 4 are completed.

## Data availability

### Underlying data

No data is associated with this article.

### Extended Data

Open Science Framework: Maternal and neonatal data collection systems in low- and middle-income countries: scoping review protocol, https://doi.org/10.17605/OSF.IO/W53JR ^15^.

This project contains the following extended data:

- Definitions of relevant terms
- List of LMICs and GAVI Countries
- Search Strategy
- GAIA project outcomes

### Reporting guidelines

Open Science Framework: PRISMA-ScR checklist for ‘Maternal and neonatal data collection systems in low- and middle-income countries: scoping review protocol’, https://doi.org/10.17605/OSF.IO/W53JR ^15^.

Data are available under the terms of the Creative Commons Zero "No rights reserved" data waiver (CC0 1.0 Public domain dedication).
