# Supplementary material for: Maternal and neonatal data collection systems in low- and middle-income countries: scoping review protocol
Source: Gates Open Res. 2020 Feb 5;4:18. [Version 1] doi: 10.12688/gatesopenres.13106.1 (PMC7199220; doi:10.12688/gatesopenres.13106.1)
Supplement: Peer review history

# Peer review: Reviewer response for version 1

**DOI:** 10.21956/gatesopenres.14271.r28775

**Published:** 2020-05-04

**Authors:** Barbara Jauregui Wurst

**Affiliations:** 1 Mérieux Foundation USA, Washington, DC, USA

This is a rigorous and well-designed protocol for a PRISMA-based scoping review of data collection systems relevant to maternal and neonatal adverse events and health outcomes to enable active pharmacovigilance surveillance of new vaccines. Studies like the one proposed are sorely needed to shed light on how little information we are collecting on these particularly vulnerable populations. 

 A growing area of interest and where there is a great need for future analysis is the use of data standards for clinical registries, perhaps a topic that could be explored or proposed in this scoping review as well. There are components of data standardization that are taken for granted in clinical trials, such as the selection of participants, data sources, and data quality, which are much more complex in public health data collection activities. Adding some questions about data storage, data quality, data cleanliness would give insight to the future use of the data being collected and its potential to be interpreted adequately, and for different sources to be consolidated to achieve greater statistical power. Data collection systems cannot work in silos anymore, and through the COVID-19 pandemic, we are seeing the devastating consequences and all the missed opportunities of having data that doesn't speak to each other. I recently supported a collaborative effort by the non-profit CDISC (www.cdisc.org) to explore the use of free open-access CDISC data standards for public health initiatives, and one of the case studies we are evaluating is precisely a New York state perinatal registry data system. I’d be happy to share the information and to put the authors of this protocol in touch with the expert leading the analysis.

Is the study design appropriate for the research question?

Yes

Is the rationale for, and objectives of, the study clearly described?

Yes

Are sufficient details of the methods provided to allow replication by others?

Yes

Are the datasets clearly presented in a useable and accessible format?

Not applicable

Reviewer Expertise:

Infectious diseases, vaccine-preventable diseases, data standards, evidence-based policy decisions.

I confirm that I have read this submission and believe that I have an appropriate level of expertise to confirm that it is of an acceptable scientific standard.

# Peer review: Reviewer response for version 1

**DOI:** 10.21956/gatesopenres.14271.r28774

**Published:** 2020-05-04

**Authors:** Alba Vilajeliu

**Affiliations:** 1 Pan American Health Organization (PAHO)/WHO Regional Office for the Americas, Washington, DC, USA

This manuscript defines a study protocol for a scoping review to identify currently active maternal and neonatal data collection systems in low- and middle-income countries (LMICs). A clear rationale is provided, and the objectives are well described. The proposed methodology is appropriate to respond to the research questions and is based on previously used frameworks. It establishes six consecutive stages and while the approach for each of the stages is well described, this reviewer would like to make some suggestions for consideration:

- Given that the detection of a rare vaccine safety signal requires a high number of pregnant women exposed to a vaccine, it is important that information systems be compatible to facilitate compilation of data. While the authors do include this as one of the characteristics to be collected, they might consider going a step further to explore whether the systems being examined allow for the transfer of electronic data to multi-country or regional pharmacovigilance networks or platforms, which would greatly increase their public health potential.
- Although the methods specify that experts will be contacted to identify unpublished information on relevant data collection systems, the protocol would benefit from further detailing how in ‘’Stage 3: Study selection’’ and ‘’Stage 4: Data extraction’’ this information about unpublished systems will be considered and included. A consultation with WHO Regional Offices could also provide insights regarding unpublished projects under development at subnational, national, and/or regional levels.
- At ‘’Stage 5: Collating, summarizing and reporting results’’, in addition to presenting the results by Gavi-supported countries vs non-Gavi supported countries, given that most of the MICs are currently not supported by Gavi, it could be relevant to stratify them based on the World Bank GNI per capita classification. This will allow differentiating between upper-middle-income countries and lower-middle-income countries.
- In reality, the existence of a maternal and neonatal active safety surveillance systems may be linked to the maturity of country maternal and neonatal immunization policies and programs. Therefore, it might be useful to map the vaccines currently recommended for pregnant women and newborns in these countries to better understand the extent of global vaccine policy uptake and its relationship to robust surveillance.

The results of this study are timely and will be relevant to informing the design and implementation of active safety electronic surveillance systems in LMICs for novel vaccines for pregnant women. It will also be important to encourage the use of Global Alignment of Immunization safety Assessment in pregnancy (GAIA project) standardized definitions that allow comparisons between different geographies.

Is the study design appropriate for the research question?

Yes

Is the rationale for, and objectives of, the study clearly described?

Yes

Are sufficient details of the methods provided to allow replication by others?

Partly

Are the datasets clearly presented in a useable and accessible format?

Not applicable

Reviewer Expertise:

Infectious disease epidemiology; vaccine research; vaccination policies; maternal and neonatal immunization; implementation of immunization programs

I confirm that I have read this submission and believe that I have an appropriate level of expertise to confirm that it is of an acceptable scientific standard.

# Peer review: Reviewer response for version 1

**DOI:** 10.21956/gatesopenres.14271.r28726

**Published:** 2020-04-14

**Authors:** Russell S. Kirby

**Affiliations:** 1 College of Public Health, University of South Florida, Tampa, FL, USA

This manuscript provides the study design for a comprehensive scoping review of existing perinatal (maternal, neonatal, or both) registries or surveillance systems in low and middle income countries. The protocol is fully developed and documented, and the authors have carefully considered what data should be collected about each data system identified. The protocol is designed to comply with or exceed the PRISMA standards for scoping reviews. This reviewer appreciates the population-based focus - while there are many facilities in LMIC countries that have perinatal registers, it is often difficult to generalize from data for a single facility. This protocol could serve as a model for future endeavors of this nature.

Is the study design appropriate for the research question?

Yes

Is the rationale for, and objectives of, the study clearly described?

Yes

Are sufficient details of the methods provided to allow replication by others?

Yes

Are the datasets clearly presented in a useable and accessible format?

Yes

Reviewer Expertise:

perinatal and pediatric epidemiology; population health science

I confirm that I have read this submission and believe that I have an appropriate level of expertise to confirm that it is of an acceptable scientific standard.
